# Variable Drug-Target Exposure, Tumor Signatures, and Combinatorial Targeted Treatment: Approaches of Personalized Medicine in Breast Cancer

**DOI:** 10.3390/jpm12060917

**Published:** 2022-06-01

**Authors:** Werner Schroth, Matthias Schwab

**Affiliations:** 1 Dr. Margarete Fischer-Bosch Institute of Clinical Pharmacology, University of Tuebingen, 70376 Tuebingen, Germany; matthias.schwab@ikp-stuttgart.de; 2 Department of Clinical Pharmacology, University of Tübingen, 69120 Tuebingen, Germany; 3 Department of Biochemistry and Pharmacy, University of Tübingen, 69120 Tuebingen, Germany

The Special Issue “Genome Research and Personalized Medicine in Breast Cancer” presents studies on personalized medicine in breast cancer, originally with a focus on genomic treatment prediction at all stages of disease. Of the five accepted articles, four dealt with hormone-receptor positive (HR+) early breast cancer, which is a reflectance of the vast abundance of this endocrine-sensitive subtype. This editorial focuses on the clinical relevance of these studies related to variable drug-target exposures, molecular tumor profiling, and the application of combinatorial targeted treatment in the adjuvant breast cancer setting. The prevalence of studies on early-stage cancer in this special issue is mirroring the emerging need for biomarker-informed treatment concepts towards the prevention and interception of metastatic disease.

The two studies by Chen et al. [1] and Almeida et al. [2] investigated the pharmacogenomic relevance of an impaired drug metabolism of tamoxifen, an antiestrogen used to prevent recurrence in premenopausal and male breast cancer, as well as in postmenopausal breast cancer of all disease stages. Tamoxifen is metabolized in the liver by a number of cytochrome-P450 enzymes, with CYP2D6 being the most relevant enzyme as it catalyzes the formation of the most abundant active metabolite endoxifen. Chen et al. [1] comprehensively investigated the relation between genetic variation in 20 CYP450 enzymes responsible for the formation of seven tamoxifen metabolites including endoxifen. They confirmed *CYP2D6* and its reduced/null activity variants as the major factor causing low endoxifen plasma concentrations. Moreover, *CYP3A4*, *CYP2C9* and the uptake transporter *SLCO1B1* may impact tamoxifen metabolism as well, supporting multi-gene diagnostic panels to predict active metabolite concentrations for personalized tamoxifen dosing. While the clinical utility of *CYP2D6* genotyping for outcome prediction is currently a matter of debate, the importance to explain the interindividual variability of endoxifen concentrations (with consequences for clinical outcome) is well accepted, as shown by the study of Almeida et al. [2]. They showed in a cohort of 149 Brazilian breast cancer patients treated with standard tamoxifen dose that endoxifen plasma concentrations below a clinical threshold of about 15 nM resulted in shorter event-free survival (EFS) compared to higher blood levels, a finding that remained significant upon adjustment for stage. However, in non-linear modeling, plasma concentrations were not significantly associated with EFS. Both studies implicate, that independent from CYP2D6 the systematic elucidation of additional confounding factors that modulate tamoxifen metabolism is mandatory for a better prediction of tamoxifen response and to guide personalized drug dosing.

Two more studies investigated the impact of tumor sequencing and/or transcriptomic analyses for an improved prognostic stratification. The study by Huang et al. [3] provided real-world evidence, that clinically relevant mutations with proven genomic/transcriptional interactions can be identified using gene signature-based risk stratification followed by targeted sequencing of actionable driver mutations. Although mutation frequencies and signature distributions may be population-specific and the study size was small, data by Huang et al. corroborates that targeted driver genes (e.g., *PIK3CA*, *SH3GLB2*) were more likely mutated in high-risk patients, pointing to the clinical perspective to select patients for advanced innovative treatment options. In a second, case/control study comprising 97 HR+/HER2- patients with or without distant metastasis at follow-up, Ni et al. [4] identified transcriptomic patterns, which are predictive for metastasis particularly in young women with early breast cancer. Using the NanoString^®^ Breast Cancer 360 panel, the study reported an enriched mTORC1 pathway in M1 patients as well as a total of 19 stratifying genes including *LRP2*, *IBSP*, and *SCUBE2* as independent prognostic factors. As the prediction of aggressive progression in young patients with hormone-sensitive subtypes is an unmet need, this study supports strategies for advanced molecular diagnostics in highly susceptible breast cancer patients to improve their therapeutic management.

Dannehl and colleagues [5] compared patient characteristics derived from a real-world setting comprising 1474 patients with data from the randomized monarchE trial combining CDK4/6 inhibition (Abemaciclib) plus endocrine treatment in early HR+/HER2- breast cancer. Surprisingly, only 50% of high-risk patients of the real-world population (eligible for CDK4/6 inhibitor treatment in theory) received chemotherapy compared to 95% of participants of the monarchE trial. Currently, the question is open whether CDK4/6 inhibition may replace adjuvant systemic chemotherapy. Thus, the study by Dannehl et al. clearly showed that a standardized risk assignment based on clinicopathological characteristics and/or molecular typing (e.g., PAM50, Oncotype) is required to select patients for systemic or targeted therapies. Moreover, biomarkers are required to predict CDK4/6-inhibition associated toxicities.
